# The ancestral architecture of the immune system in simplest animals

**DOI:** 10.3389/fimmu.2024.1529836

**Published:** 2025-01-07

**Authors:** Daria Y. Romanova, Leonid L. Moroz

**Affiliations:** ^1^ Institute of Higher Nervous Activity and Neurophysiology of RAS, Moscow, Russia; ^2^ Departments of Neuroscience and McKnight Brain Institute, University of Florida, Gainesville, FL, United States; ^3^ Whitney Laboratory for Marine Bioscience, University of Florida, St. Augustine, FL, United States

**Keywords:** Placozoa, trichoplax, long-term culturing, aging, evolution, innate immunity, NF-κB

## Introduction

~520 million years ago, the long biogeochemical history of our planet Earth resulted in a biodiversity burst. The rapid diversification of animals known as the Cambrian explosion (1); transformed planetary ecosystems with novel habitats and food chains (2, 3). However, this emergence of Paleozoic biodiversity (4) is still mysterious due to limited information about basal metazoans and their immune systems.

The representatives of the phylum Placozoa with the simplest known body plan attract the attention of ecological physiologists seeking to understand integrative responses to environmental stress, often associated with climate changes, temperature, and oxygen levels, including complex symbiotic relationships over eons (5–8). The simplicity of the cellular architecture in placozoans might correlate with a relatively small genome of 106 Mb (9). Placozoa do not have nervous and muscle systems or organs (10–13). Still, these animals have an alternative integrating system of effector cells and a number of secretory cells that, by acting on the ciliated epithelium, control locomotion (14). In this architecture, the tetraploid fiber cell type, with a significantly high DNA context, is of utmost interest since these cells potentially perform three integrative functions (15): (i) making a distributed functional network of the middle and epithelial layers (analogous to neurons), (ii) immune protection and growth control of endosymbiotic bacteria (immunity), and (iii) dynamic contractile functions (analogous to muscles) in responses to chemical environment and pH.

The combination of all three functions within one cell type is relatively rare, even in other basal metazoan lineages such as comb jellies, sponges, and cnidarians. Here, we summarize the role of systemic and cellular functions as a unique window to illuminate the origins of immune architecture in early animals using a top-down approach, from organisms to cells to molecules, including novel insights into the critical master regulators of systemic immune responses.

## Systemic and population level immunity

There is evidence for four distinct systemic immunity responses in Placozoa: (1) population aging, often associated with (2) infection; (3) resistance to radiation (next section); and (4) feeding, including interactions with pathogens and endosymbiosis. Below, we summarize the features of each of these responses.

Population aging under long-term cultivation conditions is associated with various disease-like stages (16). It includes the development of abnormal morphological changes (e.g., highly elongated body shapes) and the characteristic appearance of spherical formations on the upper epithelium with occasional rupture of the upper and middle layers. All of these can be reversed by fresh seawater and a dense substrate of microalgae as juvenilization (16). The ‘spheres’ are analogs of the encapsulation of larger foreign objects for non-specific protection. If unfavored conditions are not changed, placozoan populations are transformed into the deterioration stage of irreversible spherical formations and death without a chance of recovery.

Animals from the ‘sick’, with abundant spheres, population decrease locomotion and exploratory behaviors, as well as stop asexual reproduction. The ‘spheres’ with cavities consist of upper epithelium, fiber cells with multiple intracellular bacteria, and, probably, adjacent to the upper layer with ‘shiny’ cells containing large lipophilic inclusions with autofluorescent granules of unclear etiology but without bacteria (16). Animals can shed ‘infected cells’ encapsulated by the upper epithelium. Besides, there are morphological differences between the fiber cells of animals with endosymbionts (control) and ampicillin-treated animals (16) without bacteria. This may indicate the ability to control the reproduction of bacterial endosymbionts by the complementary activation of phagocytosis and digest bacteria as a systemic innate immunity mechanism. Combined, this dynamic organismal-level architecture provided a landscape for exploring cellular and molecular innovations in the Placozoa lineage, potentially with cross-kingdom signaling between bacterial and their hosts.

## Cellular level of immunity responses

**Figure 1:**
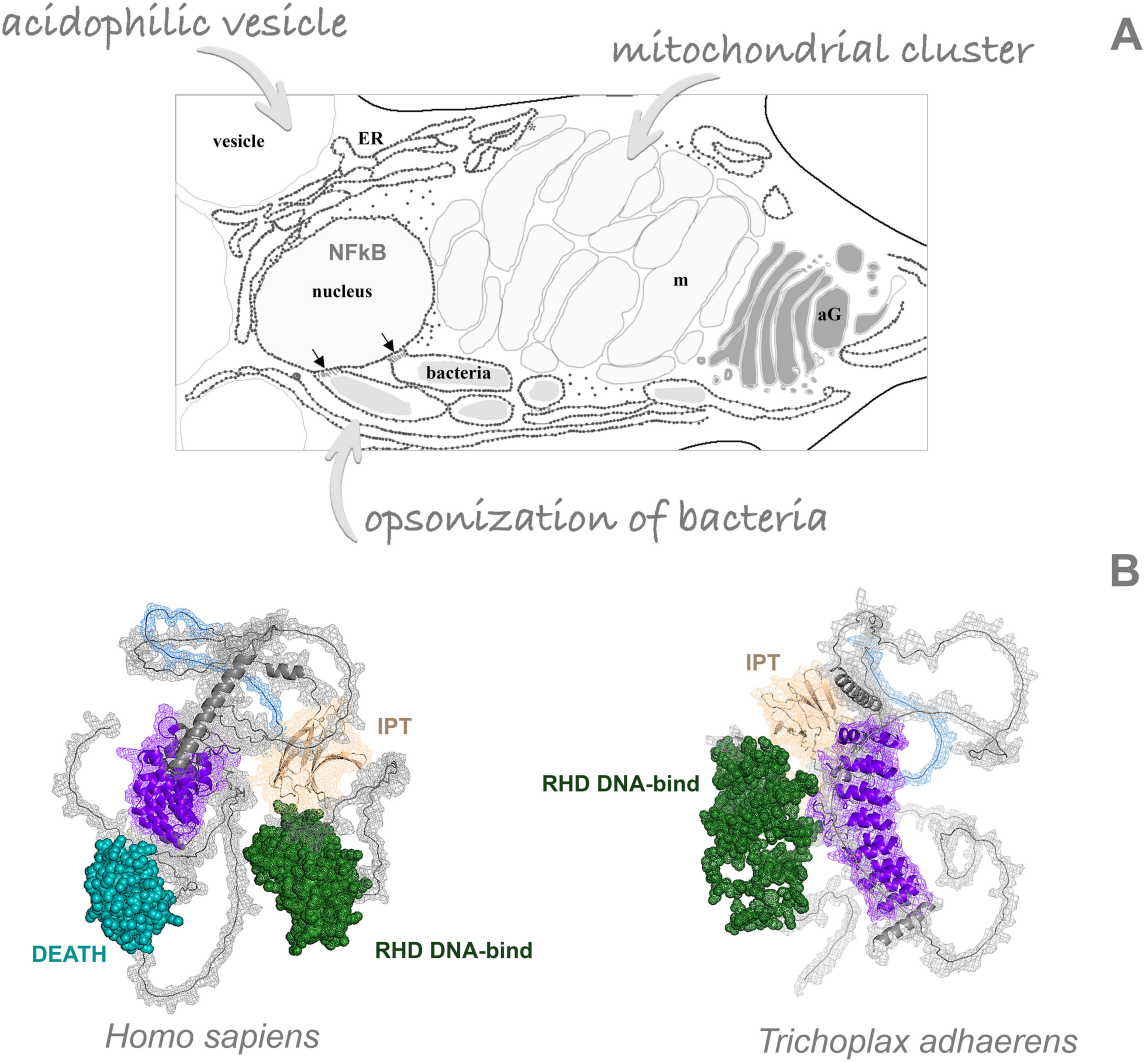
Key immunity components of Placozoa. **(A)** Multifunctional fiber cells and their intracellular architecture: nucleus, mitochondrial cluster, acidophilic vesicles and bacterial cells as endosymbionts. ER, endoplasmic reticulum; aG, Golgi apparatus. **(B)** Comparison of 3D protein models for the nuclear factor kappa B between *Homo sapiens* and *Trichoplax adherents* in. Colors: green – RHD DNA-binding domain, ultramarine – DEATH domain, wheat color – IPT domain, blue – low complexity region, deep purple – ankyrin domains. NF-kB-like protein in *Trichoplax* has one extra ankyrin domain, compared to humans, and lacks the canonical DEATH domain.

The process of shaping spherical formations involves the shiny cells of the upper epithelium [potentially releasing toxins (17)] and the middle layer cells, represented by the fiber cells (**Figure 1A**). The fiber cells represent the unique cell type associated with both integrative immune and feeding functions (18). The Gruber-Vodicka group (19) showed multiple new organelles with lysozymes in *Trichoplax* - lysozymes as widespread components of metazoan immune and digestive enzymes (20). These enzymes provide antibacterial protection by hydrolyzing the peptide glycans of bacterial cell walls and participating in the digestion process. Interestingly, it was in the fiber cells that endosymbiotic Rickettsiales bacteria were originally found in H1 and H2 haplotypes (21, 22).

Cellular immunity caused by phagocytosis (discovered by 23, 24) is the most ancient and common mechanism across eukaryotes; it includes chemotaxis, attachment of a foreign agent to the phagocyte membrane, and intracellular lysis. Chemoattractants can be signaling molecules from bacterial microorganisms, a change in the gradient of nitric oxide (NO) and related redox species (25), or a change in the amino acid composition (26). As a result of multifunctionality, fiber cell type is characterized by the presence of a massive mitochondrial cluster (13, 27, 28), which may indicate high-energy costs for metabolic processes.

In addition, fiber cells have exceptional intercellular contacts, found only in this cell type (27), which join all fiber cells into a network within the middle layer (29), and with multiple vesicles in the extracellular space (30). Thus, these are also neural-like cells that establish physiological connectivity from the middle to the dorsal layers (28–30). This ‘network’ can enable systemic immunity responses by immobilizing and consuming bacteria. Consequently, fiber cells are not fixed in their position and can be mobile as a result of chemoattraction, and be contractable.

The *Trichoplax* ventral layer is formed by cell types with adherent junctions, which tightly hold cells facing biofilm substrates. The opposite is true for the upper layer, where the cells are elastic (14). Such epithelia are evolutionary innovative structures to protect early metazoans against bacterial infection as inherent parts of innate immunity system architectures.

Opsonization, with the formation of distinct compartments, had been observed inside fiber cells (16, 28–30), and we detected bacteria in lysosomes. In some cases, bacterial cells were located in the perinuclear space (28), in close proximity to the cell nucleus. However, these are isolated cases with only 3-5% of the total number of fiber cells with bacteria. But the question remains open - why do bacteria need such close proximity to the nucleus? Is this a metabolic symbiosis?

## Molecular level of immunity

Distinctive insights into placozoan immunity were obtained by Fortunato et al. (31), who exposed *Trichoplax* to a range of X-ray doses from 143.6 to 332.5 Gy. Surprisingly, placozoans can survive and restore their populations even after 218.6 Gy exposure with immune-related adaptive morphology - the appearance of spherical formations (31), which is similar to those shown in the description ‘diseased’ in animals (16).

Transcriptome analyses after 2 hrs of exposure of animals to a dose of 218.6 Gy revealed a cascade of inducible genes coupled with genome-wide immunity responses, including genes responsible for protection against double-strand breaks, such as *RAD52*, *LIG4*, *DCLRE1C*, *RECQL5*, *XRCC6*. Other radiation-induced genes (such as *POLB*, *POLL* and *LIG3*) also control various DNA repair mechanisms (31).

Presumed immune regulatory gene networks of Toll-like receptors and scavenger receptors have been identified (22). However, these authors did not find a homolog for the nuclear factor kappa B gene. In contrast, Porifera and Cnidaria have a complete homolog of the NF-κB gene (32, 33). It raises the question of whether placozoans have a highly modified/truncated NF-κB (22) or represent an ancestral stage before the acquisition of this transcription factor in immunity mechanisms.

We performed a genome screening and found partial similarity of the human NF-κB gene with the TriadITZ_000500 gene of the H1 haplotype with a large divergence between their sequences. AlphaFold2 reliably reconstructs the three-dimensional architecture of these protein orthologs across phyla. Intriguingly, both proteins from Placozoa and Chordata are mirror images of each other with preservation in *Trichoplax* all functional domains necessary for the activity of the NF-κB (**Figure 1B**). This finding illuminates the remarkable parallelism, modularity, and perhaps convergent evolution of immunity master regulators.

## Conclusion

What might be ancestral immunity architecture? Why does the simplest, a three-layer animal of one millimeter in size, employs the astonishing diversity of immune-related genes? *Trichoplax* genome encodes 5 genes of interleukins, 4 interferons, a number of immunoglobulins, two classes of the main histocompatibility complex, secretins, tetraspannins, and other genes necessary for intercellular information transmission from cell to cell, as in the canonical chordate immune system. According to single-cell transcriptomics (34), their expression is not cluster-specific, suggesting a broad distribution of innate immunity functions across cell populations in Placozoa.

More likely, this situation reflects a great ancestral diversity of immune molecular toolkits as a result of a long history of holozoan-bacterial interactions. The early origins of multifunctional cell types with subsequent division of labor in large tetraploid fiber cells with elongated processes are morphologically and functionally similar to neurons and dendritic cells of the vertebrate immune system as well as muscle cells. These cells developed a powerful mitochondrial cluster and the largest nucleus among all *Trichoplax* cell types. Combined, these traits are associated with the omnipresence of bacteria both as food sources and pathogens with a dual role of bioenergetically demanding phagocytosis. Bacteria encapsulation might also be an ancestral immune strategy by making ‘shells’ of the ‘friend-or-foe’ recognition system with the recruitment of opsonin-like genes (35–38).

Finally, fiber cells might be viewed as functional analogs of interneurons, with distributed connectivity and their localization uniting all cell layers. They form a highly distributed ‘network’ created by long processes of fiber cells for more efficient and rapid intercellular communications, integrating neural, hormonal, and contractive integrative systems. It would not be surprising if fiber cells were excitable with low-frequency oscillations (e.g., 100-150 ms) capable of secretion and chemical connectivity (30).

In sum, the *multifunctionality of fiber cells* can be the main operational module of the immune system for Placozoa and likely an example of exaptation for other emerging properties as dynamic links between immune and neural functions, which are responsible for organism-level integration. We would not be surprised if fiber cells could have epigenetic machinery for immune ‘cellular memory’ as the predecessor of acquired immunity.
